# Supplementary material for: Elucidating the Structural Impacts of Protein InDels
Source: Biomolecules. 2022 Oct 7;12(10):1435. doi: 10.3390/biom12101435 (PMC9599607; doi:10.3390/biom12101435)
Supplement: Supplementary file 1 [file biomolecules-12-01435-s001.zip › biomolecules-1889019-supplementary.pdf]

Elucidating the Structural Impacts of Protein InDels – Supplementary Material

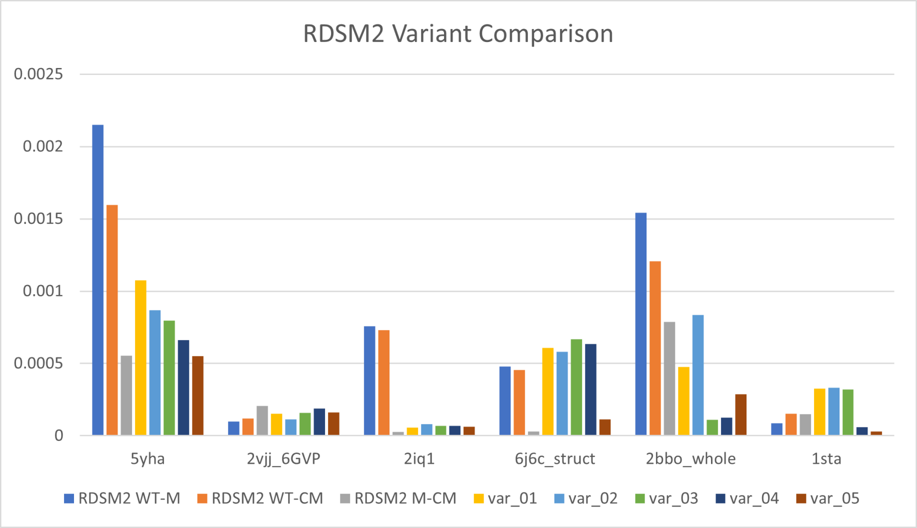

Figure S1: Comparison of the RDSM2 scores of both our original method, and 5 additional computationally generated mutants compared with our computa-tionally generated mutant.

Table S1: RDSM2, RDSM3, and TLCCS between each of the computationally generated variants and their associated computationally generated mutant.

Variant	RDSM2	RDSM3	TLCCS
5yha var01	0.0011	0.00078	0.24
5yha var02	0.00087	0.00073	0.34
5yha var03	0.00080	0.00052	0.37
5yha var04	0.00066	0.00053	0.39
5yha var05	0.00055	0.00031	0.48
2vjj 6GVP var01	0.00015	0.00011	0.86
2vjj 6GVP var02	0.00011	0.00011	0.87
2vjj 6GVP var03	0.00016	0.00012	0.87
2vjj 6GVP var04	0.00019	0.00013	0.83
2vjj 6GVP var05	0.00016	0.00011	0.82
2iq1 var01	0.000057	0.000013	0.86
2iq1 var02	0.000082	0.000024	0.83
2iq1 var03	0.000070	0.000020	0.83
2iq1 var04	0.000070	0.000019	0.87
2iq1 var05	0.000062	0.000016	0.90
6j6c struct var01	0.00061	0.00021	0.43
6j6c struct var02	0.00058	0.00019	0.44
6j6c struct var03	0.00067	0.00027	0.36
6j6c struct var04	0.00064	0.00025	0.38
6j6c struct var05	0.00011	0.00011	0.84
2bbo whole var01	0.00048	0.00040	0.58
2bbo whole var02	0.00084	0.00060	0.47
2bbo whole var03	0.00011	0.00012	0.74
2bbo whole var04	0.00013	0.00015	0.67
2bbo whole var05	0.00029	0.00017	0.73
1sta var01	0.00033	0.000054	0.77
1sta var02	0.00033	0.000057	0.77
1sta var03	0.00032	0.000052	0.76
1sta var04	0.000059	0.000022	0.84
1sta var05	0.000030	0.000012	0.83

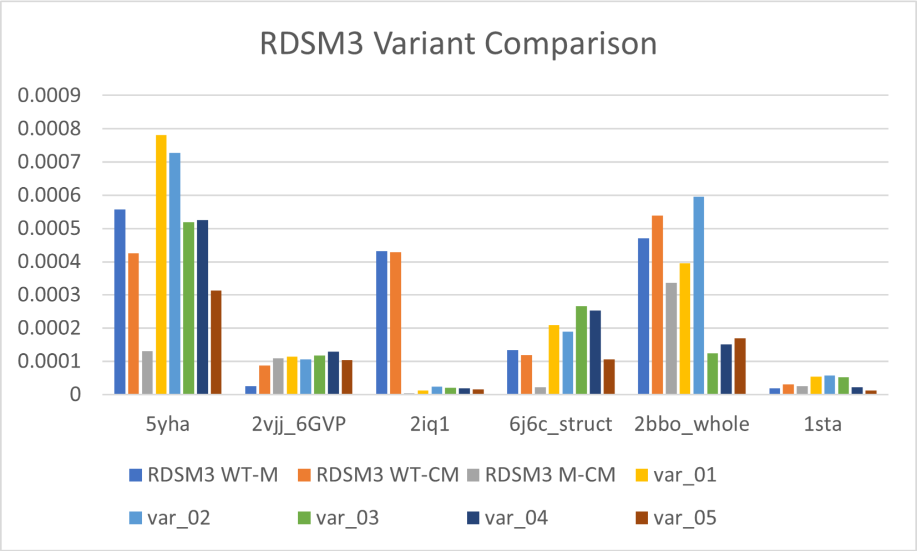

Figure S2: Comparison of the RDSM3 scores of both our original method, and 5 additional computationally generated mutants compared with our computa-tionally generated mutant.
